# The Role of Histone Post-Translational Modifications in Merkel Cell Carcinoma

**DOI:** 10.3389/fonc.2022.832047

**Published:** 2022-03-08

**Authors:** Chiara Mazziotta, Carmen Lanzillotti, Roberta Gafà, Antoine Touzé, Marie-Alice Durand, Fernanda Martini, John Charles Rotondo

**Affiliations:** ^1^ Department of Medical Sciences, University of Ferrara, Ferrara, Italy; ^2^ Center for Studies on Gender Medicine, Department of Medical Sciences, University of Ferrara, Ferrara, Italy; ^3^ Department of Translational Medicine and for Romagna, University of Ferrara, Ferrara, Italy; ^4^ ISP “Biologie des infections à polyomavirus” Team, UMR INRA 1282, University of Tours, Tours, France; ^5^ Laboratory for Technologies of Advanced Therapies (LTTA), University of Ferrara, Ferrara, Italy

**Keywords:** Merkel cell carcinoma, histone post-translational modifications, epigenetics, Merkel cell polyomavirus, acetylation, methylation, phosphorylation, histone deacetylase inhibitors

## Abstract

Merkel Cell Carcinoma (MCC) is a rare but highly aggressive form of non–melanoma skin cancer whose 5-year survival rate is 63%. Merkel cell polyomavirus (MCPyV), a small DNA tumor virus, is the etiological agent of MCC. Although representing a small proportion of MCC cases, MCPyV-negative MCCs have also been identified. The role of epigenetic mechanisms, including histone post-translational modifications (PTMs) in MCC, have been only partially determined. This review aims to describe the most recent progress on PTMs and their regulative factors in the context of MCC onset/development, providing an overview of current findings on both MCC subtypes. An outline of current knowledge on the potential employment of PTMs and related factors as diagnostic and prognostic markers, as well as novel treatment strategies targeting the reversibility of PTMs for MCC therapy is provided. Recent research shows that PTMs are emerging as important epigenetic players involved in MCC onset/development, and therefore may show a potential clinical significance. Deeper and integrated knowledge of currently known PTM dysregulations is of paramount importance in order to understand the molecular basis of MCC and improve the diagnosis, prognosis, and therapeutic options for this deadly tumor.

## Introduction

Merkel cell carcinoma (MCC) is a rare, but aggressive non–melanoma skin cancer (1–3). Although similar presentation and prognosis, two different MCC aetiologies have been identified. The first, which accounts for the highest proportion (4–8), ~80% of cases, is caused by a DNA tumor virus belonging to the *polyomaviridae* family, i.e., Merkel cell polyomavirus (MCPyV) (2, 9). The expression of the two viral oncoproteins truncated large T antigen (tLT) and small T antigen (sT), alongside the integration of the MCPyV DNA into the host genome, are the main events for MCPyV-positive MCC (MCCP) onset (9–12). In MCCP cells, LT expression leads to cell proliferation maintenance (13), while sT is required for cell transformation and survival (14). The second MCC form MCPyV-negative (MCCN), is caused by high levels of UV-induced tumorigenic point mutations (10, 15, 16). Despite MCCN tumor having a high mutational burden, MCCP harbours a 100-fold lower mutational load (17). According to the Surveillance, Epidemiology, and End Results (SEER) Program, the MCC 5-year survival rate is 76, 53 and 19% for localized, regional and distant disease, respectively, with an overall combined rate of 63% (18–21). MCC global incidence ranges 0.1-1.6 cases/100,000 individuals (22). UV light exposure in fair-skinned people, possibly due to occupational sunlight exposure (23), anti-viral/-tumor immunological decline (6, 24, 25), and old age are considered as MCC risk factors (26). MCC onset risk increases in patients under iatrogenic immunosuppression (9, 27–29). At primary diagnosis, ~30% of MCC patients present adjacent metastasis and/or lymph node metastases (26), while current therapies are poor. The most effective MCC therapy is cisplatin and etoposide cytotoxic chemotherapy. However, this therapeutic approach is limited by mean progression-free survival being estimated at ~3 months (30). Moreover, some patients are not responders and/or develop therapy resistance. Surgery and radiation therapies are adopted for local and nodal MCC (31, 32), while systemic therapy is preferred for extensive, metastatic, and recurrent tumor. Even when early diagnosed, patients may not be eligible for surgery/radiotherapy due to other comorbidities. The use of Programmed cell death protein 1 (PD-1) and Programmed death-ligand 1 (PD-L1) inhibitors seems to be an effective therapeutic approach (33–36). Novel therapies are also under evaluation (31, 36–38).

The role of histone post-translational modifications (PTMs) in MCC has been remarked upon (2). PTMs, DNA methylation and microRNAs (miRNAs), are fundamental epigenetic mechanisms for controlling gene expression (39–42). These mechanisms collectively determine the accessibility of gene promoters to RNA polymerase II and transcription factors. Histone octamer core complex is composed of two copies each histone protein H2A, H2B, H3, and H4 and is surrounded by 147 bp of DNA to form a nucleosome, the main structural chromatin unit (43). PTMs mostly occur on histone N-Terminal regions (histone tails) protruding from the histone core. These modifications regulate the expression of genes by chromatin remodelling. The combination of single/multiple PTMs and their regulatory role on gene expression are referred to as histone code (44). These processes typically occur in concert with DNA methylation (45–47). The main PTMs are acetylation, methylation, and phosphorylation. Less well-understood PTMs include glycosylation, ubiquitylation, sumoylation, carbonylation and ribosylation (48). The combination of different PTMs can modulate gene expression. Impairment of both PTMs and modifying enzymes has been associated with various diseases, including cancer (2, 49). However, despite large number of PTMs and modifying enzymes being identified, a functional understanding of most is still yet to be gained.

Growing evidence indicates that PTM dysregulation play a role in MCC (2, 50, 51). Studies aimed at epigenetically characterizing MCC have reported data on acetylation, methylation, and phosphorylation, alongside histone modifying enzymes (2). However, the relationship between these dysregulations and MCC onset, progression and metastasis have only been covered partially, while the clinical application of most remains to be determined. Moreover, how MCPyV oncoproteins are capable of dysregulating PTMs in MCCP onset is unclear. Indeed, unlike other DNA tumor viruses (52–56) whose oncogenic activity has been linked (57, 58), and demonstrated as functionally dysregulating a variety of PTMs (59, 60), little information has yet been reported for MCPyV. Notably, PTM dysregulation offers both diagnostic and prognostic potential in the clinic and represents a therapeutic target in a large variety of tumors (57).

This review is addressed at collecting and summarizing the state of the art on PTMs and regulative factors whose dysregulation has been assumed to play a role in MCC. A description of current knowledge on the potential usage of PTMs and regulative factors as diagnostic and prognostic tools, as well as targets for MCC therapy is also provided.

## Histone Post-Translational Modifications and Merkel Cell Carcinoma

### Histone Acetylation

Histone acetylation occurs on lysine residues and leads to chromatin relaxation by creating repulsive forces with the negatively charged DNA. This process makes the DNA accessible to the transcription factors, ultimately leading to gene expression (61–63). Histone deacetylation generally induces chromatin condensation while being linked to gene silencing (64). Histone acetylation is mediated by Histone Acetyltransferases (HATs), while Histone Deacetylases (HDACs) catalyze deacetylation reactions (65).

**Figure 1:**
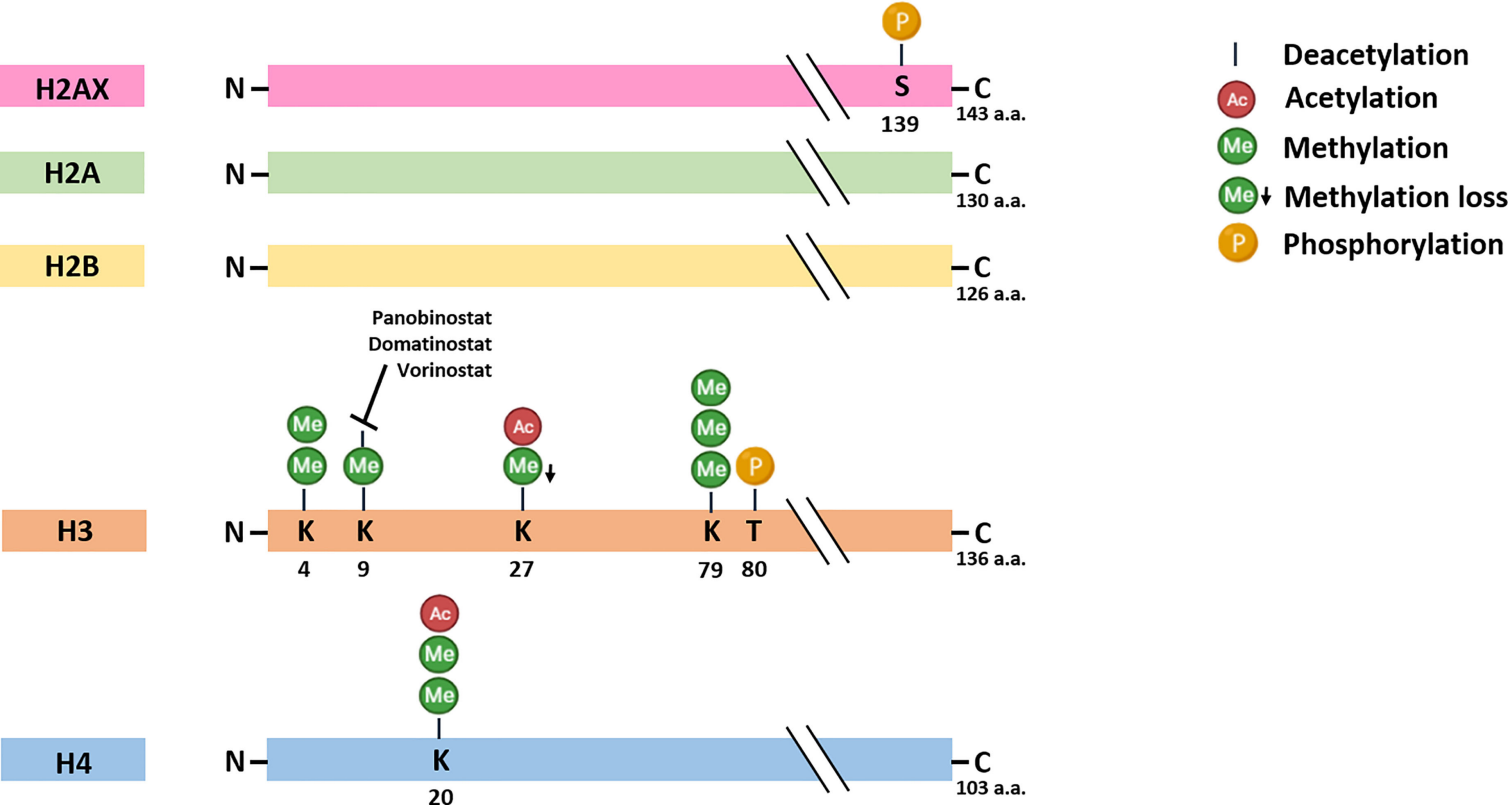
Histone post-translational modifications in Merkel cell carcinoma. The specific modifications and amino acids (a.a.) residues are reported. Histone deacetylase inhibitors Panobinostat, Domatinostat and Vorinostat has been found to restore histone acetylation *in vitro* in MCC cell lines, thereby promoting gene expression. S, Serine; K, Lysine; T, Threonine.

Histone acetylation dysregulation is involved in MCC host immune-surveillance escape. As other tumors (25, 66–68), MCC present strong immuno-selective pressures and, thus, arises and progress when developing efficient immune escape mechanisms. One strategy provides the improper recognition by T cells owing to the downregulation of major histocompatibility complex (MHC) class-I on tumor cell surfaces. Indeed, MHC class-I encodes for surface receptors which are essential for the adaptive immune system (66, 69, 70). Ritter and colleagues reported on the loss of MHC class I chain-related protein (MIC) A and B expression in MCCP cells (71). MICs are polymorphic proteins whose expression is induced upon cell transformation and act as kill me signals for natural killer cells, which are therefore activated against tumor cells during the innate immune response. This loss has been afterwards reported as a consequence of H3K9 deacetylation nearby the MIC promoter (71). MIC expression can also be epigenetically restored by pharmacological treatment with the HDAC inhibitor (iHDAC) Vorinostat (**Figure 1**) (71). Additional cell surface receptors encoded by MHC locus, i.e., HLA class-I complex, have been reported as down-regulated MCC tissues with unknown MCPyV-positivity and in MCCP cell lines (69). This down-regulation has been related to the impaired expression of key components of antigen processing machinery (APM), including low-molecular-weight protein (LMP) 2 and LMP7, as well as a transporter associated with Antigen Processing 1 and 2. *In vitro/in vivo* data also indicate that HLA class-I APM down-regulation is attributable to a dysregulated H3K9 deacetylation in proximity to APM gene promoters, whilst Vorinostat has been reported as capable of restoring their expression (69). The epigenetic re-expression of HLA class-I on the surfaces of MCC cells has also been demonstrated with Domatinostat, an orally available iHDAC (**Figure 1**) (72). Following pharmacological treatments, RNA sequencing and functional experiments indicated a distinct gene expression signature for antigen processing and presentation, cell-cycle arrest and apoptosis. Therefore, the re-expression of HLA class-I by Domatinostat might favour MCC cell identification and eradication by the anti-tumor immune response (72).

Inhibitory receptors expression on tumor-targeted immune cells and tumor cells surfaces can be considered as another MCC immune escape mechanism. During cell transformation, the interaction between PD-1 and PD-L1 expressed on the surface of activated T cells and tumor cells, respectively, reduces T-cell function and prevents the immune system from acting against tumor cells (73). Although PD-1/PD-L1 blockade therapy is promising for cancer treatment (74), a fraction of patients are either non-responders or develop resistance (75, 76). To circumvent these negative responses, therapies combining PD-1/PD-L1 blockers and iHDACs have been developed for MCC. A Phase II clinical trial (NCT04393753) is currently ongoing to investigate the efficacy/safety of the anti-PD-L1 antibody avelumab in combination with domatinostat in MCC patients. Furthermore, the iHDAC Panobinostat has been administered to a small group of PD-1/PD-L1 non-responder metastatic MCC patients (77). The pharmacological treatment of two patients with Panobinostat was reported as inducing a restored HLA class-I expression and an increase in CD8^+^ T-cell infiltration in tumor tissues. These data suggest that iHDACs might be helpful in overcoming HLA class-I expression loss in PD-1/PD-L1 non-responder patients (77). In summary, histone acetylation dysregulation in chief immune system players represents a key mechanism whereby MCC can escape the anti-tumor response, while expression restoration in immune regulatory complexes by iHDACs epigenetic priming might be a helpful therapeutic approach based on boosting adaptive immune responses (38).

Histone acetylation impairment in MCC encompasses the dysregulation of proto-oncogene *c-Myc* (50). In MCC tissues/cells with unknown MCPyV positivity and in MCCP cell lines, c-Myc overexpression has been linked to acetylated lysine 27 enrichment at histone H3 (H3K27Ac) in an enhancer proximal to the *c-Myc* promoter. At the same time, high occupancy of Bromodomain protein 4 (BRD4), a gene expression regulator which binds to H3K27Ac, has also been reported. Also, *in vitro* treatment with bromodomain and extra-terminal (BET) inhibitors (iBETs), a class of drugs that reversibly bind to BET family proteins, comprising BRD4, depleted BRD4 occupancy at the *c-Myc* enhancer and induced c-Myc down-regulation (50). This evidence underlines not only that both H3K27Ac and BRDs play a role in MCC, but also that iHDAC and iBET combination therapy should be considered as a novel therapeutic approach.

### Histone Methylation

Histone methylation provides the addition of a methyl group on lysine and arginine residues, which can undergo mono-/di-/trimethylation, and mono-/dimethylation, respectively. Enzymes mediating histone methylation and demethylation reactions are histone methyltransferases (HMTs) and demethylases (HDMs), respectively (78). Histone methylation can be associated with either transcriptional repression or activation (79, 80). Important HMT/HDM enzymes are: (i) Enhancer of zeste homolog 2 (EZH2) which is the catalytic subunit of the Polycomb Repressive Complex 2 (PRC2); the complex methylates lysine 27 in histone H3 (H3K27) (81), a repressive mark on tumor suppressor gene promoters during tumorigenesis (82, 83); (ii) Lysine-specific demethylase 1 (LSD1) which removes the mono-/di- methylation marks in lysine 4 and 9 of histone H3 (H3K4/9) (84, 85). H3K9 tri-methylation is almost exclusively localized in pericentric heterochromatin, repetitive elements and non-coding portions of the genome (86), while H3K27 tri-methylation is mainly associated with gene silencing and can be found in genes rich areas (87, 88). Methylated H3K4 has been found in transcriptionally active euchromatic regions (89).

Significant attention has been paid to histone methylation and particularly to LSD1 (17, 90), with the aim of developing novel MCC therapies (**Figure 1**) (17, 91, 92). During MCCP onset, sT can interact with several factors, such as MYCL and MAX. Both factors are recruited to the EP400 subunit (or p400) of the histone acetyltransferase complex by sT (90). Improper complex activation leads to LSD1 overexpression, thereby favouring MCC growth *in vitro/in vivo* (91). Consistently, LSD1 inhibition *in vitro* can induce cell cycle arrest and apoptosis in MCCP cells, while activating a gene expression signature which resembles normal Merkel cells; tumor growth inhibition has also been shown *in vivo* (17). LSD1 inhibition could therefore be considered as an additional MCC therapeutic option (17, 91–93).

The role played by PRC2 and its functional enzymatic component EZH2 in MCC progression and metastasis has been remarked upon. Increased EZH2 expression has been reported as associated with both MCC progression and poor prognosis (81). Immunohistochemistry (IHC) experiments conducted on MCCP and MCCN tissues, including primary tumors, as well as lymph node, in-transit and distant metastases, reported about half specimens as exhibiting strong/moderate EZH2 expression. Weak EZH2 expression in the primary tumor, but not nodal metastases, has been correlated with improved prognosis. High EZH2 expression levels have also been reported in a set of MCC tissues, with no differences according to MCPyV presence (94). Although H3K27me3 has not been studied, these papers underlined the prognostic value of EZH2 in MCC, thus suggesting that this HMT might be a potential target for MCC treatment.

Additional studies have examined H3K27me3 in different primary/metastatic MCC tissue specimens, such as: (i) MCCP and MCCN tumors with pure histological features, including primary and metastatic lesions, as well as a small number of combined squamous and neuroendocrine carcinomas (51); (ii) different tumor types, including MCC (95); (iii) MCC tissues stratified according to MCPyV status and morphological type (49). The first study described reduced H3K27me3 expression in MCCPs and in MCCs with pure histologic features (51). The second reported H3K27me3 loss in 90% of MCCs with unknown MCPyV positivity, while all MCCs were H3K27me2-positive (95). The third reported low H3K27me3 levels in MCCN compared to MCCP tumors, and in particular in MCCNs combined with squamous cell carcinoma than in MCCP/N pure tumors or pure histologic MCCs (regardless of MCPyV status). Following prospective analyses indicated a lack of correlation between low H3K27me3 and MCC patient outcome, thus excluding a prognostic role for this epigenetic mark (49). Considering these data, the loss of H3K27me2/me3 marks in MCC might be attributable to an impairment in PRC2 activity (96). However, neither PRC2 expression nor activity has been evaluated in these studies. H3K27me2/me3 modifications might potentially be due to PRC2 levels/activity changes. However, a link between PRC2 and H3K27me2/me3 upon MCC has not been determined. In this context, whether PRC2 and its epigenetic marks play a role in MCC onset is yet to be verified with further studies.

The important role of histone methyltransferase PRDM8 in MCCN development has recently been highlighted (97). High PRDM8 mRNA levels have been reported in MCCN cell lines, while PRDM8 overexpression has been found as linked to an increase in H3 lysine 9 methylation (H3K9me) global levels in MCCN specimens. Notably, the same study also identified *miR-20a-5p* as an upstream PRDM8 regulator, pointing to its involvement in MCC tumorigenesis as a tumor suppressor miRNA. Taken together, these findings provide insight into the role of PRDM8 and histone methylation in MCC (97).

### Histone Phosphorylation

Histone phosphorylation provides the addition of phosphate groups to serine, tyrosine and/or threonine residues (98–100). Phosphatases and kinases are the two enzyme classes known to remove and include the phosphate groups, respectively (101, 102). Although being somewhat less well-understood than acetylation and methylation, phosphorylation seems to be linked to positive gene regulation, as leading to chromatin relaxation (101, 102).

The involvement of histone phosphorylation in MCC has been investigated in studies focused in both histone methylation and phosphorylation marks. H3 lysine 79 trimethylation/H3 threonine 80 phosphorylation (H3K79me3T80ph, or H3KT) and H3 phosphorylation (PHH3) marks, which are both expressed during G2/M phase progression, have been assessed as prognostic markers for MCC in relation to mitotic figures and G2+ tumor nuclei. The latter are both proliferation indicators. Despite the limited number of MCC patients enrolled in the study, H3KT/PHH3 marks, evaluated by IHC in MCC tissues with unknown MCPyV positivity, had prognostic significance in stratifying patient risk in relation to proliferative rates (103). The role of histone methylation and phosphorylation in MCCP during MCPyV sT-induced DNA damage response (DDR) pathway activation has also been reported (104). MCPyV sT overexpression has been described as functionally inducing (i) H2AX phosphorylation *via* ataxia telangiectasia mutant (ATM) activation, which is a crucial upstream kinase for H2AX phosphorylation; (ii) dimethylation of H3 lysine 4 (H3K4me2) and H4 lysine 20 (H4K20me2). Moreover, phosphorylation of other DDR/ATM downstream proteins has also been described. These findings cumulatively suggest the contribution of histone methylation and phosphorylation in MCPyV sT-induced DDR pathway activation upon MCCP onset (104).

## Histone Variants and Merkel Cell Carcinoma

Mass spectrometry analyses conducted on MCCP cell lines compared to a control epithelial cell line reported a total of 5 different histone families with 15 different subfamily members, including H2A type 1-H as being differentially expressed (105). Although these histones have not been investigated epigenetically, the study highlighted the role of histone variants in MCCP. Further studies might extend these proteome data towards an epigenetic point of view.

## Discussion and Future Perspectives

This review summarizes and provides insights into PTMs and related factors being assessed as linked to MCC (**Figure 1**). While emerging evidence indicates that PTM dysregulations are involved in MCC, the clinical application of most has only been limitingly demonstrated. The impairment of histone acetylation (50, 69, 71), methylation (49, 51, 95) and phosphorylation marks (103, 104) as well as histone modifying enzymes, including PCR2/EZH2 (81, 94), LSD1 (17, 90, 91) and PRDM8 (97), have been reported as playing a role in MCC. From a therapeutic point of view, promising data have been obtained with iHDAC inhibitors, such as Vorinostat, Domatinostat and Panobinostat (17, 69, 71, 72, 77), as well as with LSD1 inhibitors (17). Moreover, studies conducted with Vorinostat and LSD1 inhibitors have been conducted both *in vitro* with MCC cells, and *in vivo* with animal models, thus underling the reliability of these antitumor compounds (17, 69, 91). The clinical application of iHDACs has also been demonstrated with MCC patients under combination therapy with PD-1/PD-L1 inhibitors in one study and in one phase II clinical trial (NCT04393753) (77). Contrariwise, a few studies questioning PTMs as MCC diagnostic/prognostic markers, comprising histone methylation (49, 51, 95) and simultaneous methylation and phosphorylation (103), have been reported. Further studies should be performed to identify novel PTMs as MCC diagnostic/prognostic markers.

As hypothesized in the abovementioned studies, the dysregulation of epigenetic mechanisms plays a role in MCC to some extent and therefore represent potential clinical significance. The identification of novel PTMs may improve early MCC diagnosis and therapy monitoring. MCC onset, progression and metastasis is often rapid (32) and early tumor identification, as well as expeditious diagnostic workup and therapy initiation are crucial. Thus, research for novel PTMs to be employed in the clinic should be a priority (106). Since MCC is considered an aggressive and deadly tumor, there is an urgent need to identify novel effective therapies for its management. As the malignant behaviour of MCC cells can be reverted with iHDACs (17, 69, 71, 72, 77), combination multidrug therapies with hypomethylating agents should be considered (45, 107, 108), as has successfully been demonstrated in treating other tumors (109–111). We therefore recommend further rigorous preclinical/clinical studies in this direction (112).

In conclusion, while promising data have been obtained for PTMs and modifying enzymes, the clinical application of most is still to be verified. Further studies are needed for identifying novel PTMs that could be employed as clinical tools. Gaining a deeper understanding of PTM dysregulations is of paramount importance for understanding the molecular basis of MCC and improving the diagnosis, prognosis, and therapeutic options for this tumor.

## Author Contributions

CM wrote the first draft of the manuscript. M-AD and RG selected the literature and revised the manuscript draft. CL designed the figures. JCR, AT, and FM organized and supervised the work, and corrected the manuscript draft. CM and JCR wrote the final version of the manuscript. All authors contributed to the article and approved the submitted version.

## Funding

This work was supported, in part, by grants MFAG 21956 (to JCR), from the Associazione Italiana per la Ricerca sul Cancro (AIRC), Milan, Italy, and University of Ferrara, FAR grants 2021 (to Fernanda Martini). CM is supported by a AIRC fellowship for Italy (ID: 26829).

## Conflict of Interest

The authors declare that the research was conducted in the absence of any commercial or financial relationships that could be construed as a potential conflict of interest.

## Publisher’s Note

All claims expressed in this article are solely those of the authors and do not necessarily represent those of their affiliated organizations, or those of the publisher, the editors and the reviewers. Any product that may be evaluated in this article, or claim that may be made by its manufacturer, is not guaranteed or endorsed by the publisher.
